# Naked-Eye Detection of Glucose in Saliva with Bienzymatic Paper-Based Sensor

**DOI:** 10.3390/s18041071

**Published:** 2018-04-03

**Authors:** Luis A. Santana-Jiménez, Alfredo Márquez-Lucero, Velia Osuna, Ivan Estrada-Moreno, Rocio B. Dominguez

**Affiliations:** 1 Center for Research in Advanced Materials (CIMAV S.C.), Miguel de Cervantes 120, 31136 Chihuahua, Mexico; luis.santana.jimenez@gmail.com; 2 CIMAV S.C., Ejido Arroyo Seco, 34147 Durango, Mexico; alfredo.marquez@cimav.edu.mx; 3 CONACyT-CIMAV S.C., Miguel de Cervantes 120, 31136 Chihuahua, Mexico; velia.osuna@cimav.edu.mx (V.O.); ivan.estrada@cimav.edu.mx (I.E.-M.)

**Keywords:** paper-based sensor, saliva, naked-eye, glucose, low-cost

## Abstract

The high incidence of Diabetes Mellitus in low-income regions has promoted the development of low-cost alternatives to replace blood-based procedures. In this work, we present a bienzymatic paper-based sensor suitable for the naked-eye detection of glucose in saliva samples. The sensor was obtained by a stamping procedure and modified with chitosan to improve the colorimetric readout. The bienzymatic reaction of GOx-HRP coupled with 2,4,6-tribromo-3-hydroxy benzoic acid was applied for the detection of glucose within a range from 0 to 180 mgdL^−1^ in buffer and artificial saliva solutions. The visual readout was perceived by the naked eye and registered with an office scanner to evaluate the analytical performance. The results showed a limit of detection of 0.37 mgdL^−1^ (S/N = 3) with an R.S.D. of 1.69% and a linear range from 1 to 22.5 mgdL^−1^ with an R^2^ of 0.99235. The analysis of human saliva samples was performed without pre-processing, achieving recoveries from 92 to 114%. The naked-eye detection was evaluated under two different light settings, showing average recoveries of 108.58 and 90.65% for standard and low illumination. The proposed device showed potential for easy-to-use, sensitive, low-cost, fast, and device-free detection of salivary glucose suitable for untrained personnel operation and limited facilities.

## 1. Introduction

The high incidence of Diabetes Mellitus (DM) in low- and middle-income countries represents a major concern given the a lack of resources in infrastructure and trained personnel of these regions [1,2,3]. This has motivated a demand for low-cost, reliable, and easy-to-use platforms for monitoring and diagnosis, which should follow the ASSURED criteria (affordable, sensitive, specific, user friendly, rapid and robust, equipment free, and deliverable) in order to provide results even in the most limited facilities [4]. Paper-based sensors are analytical tools created by delimiting hydrophilic zones of cellulose substrates with hydrophobic reagents. The designed patterns can be used as microchannels to transport samples and reagents or as detection zones which can be functionalized to perform easy detection. Paper-based sensors can fulfill most of the requirements of the ASSURED criteria as well as present some relevant advantages such as high surface-to-volume ratio, biocompatibility, porosity, low-cost, biodegradability, lightness, and flexibility [5,6,7]. The fabrication of the patterning design can be achieved at a high resolution with manufacturing methods such as wax printing, screen printing, photolithography, and flexography [6,8]. However, manual techniques such as blade coating, manual drawing, and stamping allow rapid prototyping with minimal investment of equipment and good repeatability in fabrication [8,9]. The readout of paper-based sensors can be performed with colorimetric and electrochemical transducers, but the colorimetric techniques offer advantages such as sensitivity, stability, and simplicity of detection through direct visualization as opposed to the readers needed for electrochemical devices [10,11,12]. However, a major shortcoming of colorimetric transduction is the irregular spot produced by the elution of reagents in the detection zone, which can negatively impact the sensitivity of the device [13]. To improve this, agents with good properties for film creation such as gelatin and chitosan have been applied to modify the detection zone and obtain a homogeneous outcome [13,14,15].

The high incidence of DM worldwide has promoted an unprecedented interest in the development of [16] painless glucose detection methods with easily accessible body fluids such as urine, tears, sweat, and saliva [17,18,19]. In this sense, the studied correlation between glucose content in saliva and blood has been proposed as a potential non-invasive method for DM monitoring, mainly due to the advantages of this sample such as easy and economical collection, a relation with the physiological state of the body, and non-invasiveness [18,20,21]. However, the low glucose concentration of saliva compared with blood glucose prevents the application of commercial glucose self-monitoring technology for non-invasive detection. Thus, a variety of devices to address salivary glucose detection have been proposed, such as Single Walled Carbon Nanotubes (SWCNT)/chitosan/Au Nanoparticles/Glucose oxidase (GOx) nanobiosensors [22], CuO nanoneedle/graphene/carbon nanofiber non-enzymatic sensors [23], reduced graphene oxide (RGO)/chitosan/nafion/GOx electrochemical sensors [16], and RGO/carbon dots photoluminescent assays [24]. The colorimetric detection of glucose in body fluid samples have been performed with a test strip of GOx co-immobilized with methyl red dye [25], a ZnFe_2_O_4_ magnetic nanoparticles assay [26], and a GOx/Horseradich Peroxidase (HRP)/chitosan paper device [14]. Recently, 2,4,6-tribromo-3-hydroxy benzoic acid (TBHBA) was also proposed as a chromophore alternative for paper-based sensors mainly due to its water solubility, high absorptivity, and stability from the produced quinone-imine dye, as well as its strong attachment to cellulose due to its positive charge [15,27].

Since simple detection methods for body fluids, like saliva, are highly desirable, in this work we present the development of a paper-based, low-cost, and reliable platform suitable for the direct “naked-eye” detection of salivary glucose. The device was created by delimiting microzones with a manual stamping process and chitosan modification in order to obtain a homogeneous spot. This biocompatible zone was used to perform the bienzymatic GOx-HRP reaction along with TBHBA as a chromophore. The visual result was correlated with the glucose concentration by the naked eye and with image processing software to evaluate the analytical performance. Stability, selectivity, and non-processed real sample tests showed the potential applicability of the device for glucose detection in saliva samples with minimal investment and an easy-to-use method.

## 2. Materials and Methods

### 2.1. Reagents

GOx type X-S from *Aspergillus niger* (128.2 U/mg), HRP type II (210 U/mg), TBHBA 97% (CAS 14348-40-4), chitosan powder (CAS 9012-76-4), d-(+)-Glucose (CAS 50-99-7), 4-aminoantipyrine (CAS 83-07-08), paraffin (CAS 8002-74-2), acetic acid (CAS 64-19-7), and # 40 Whatman^TM^ filter paper were purchased from (Sigma-Aldrich, Toluca, Mexico). Phosphate buffer (PB) at 100 mM, pH 7.0 was prepared from potassium phosphate monobasic and sodium phosphate dibasic, both obtained from Sigma-Aldrich. Phosphate buffer (100 mM, pH 6.0 at 20 °C) was prepared from potassium phosphate monobasic (CAS 7778-77-0). Artificial saliva was obtained from Viarden (Mexico City, Mexico). Working solutions of glucose were prepared from a stock solution of 180 mg/dL in PBS and artificial saliva.

### 2.2. Instruments and Software

The modification of the detection zone was evaluated by contact angle measurements using FTA32 (First Ten Armstrong, Portsmouth, PA, USA) equipment. To register the color intensity, a multifunctional HP MFP M1176n (Hewlett-Packard, Palo Alto, CA, USA) office scanner at 1200 dpi was used. The intensity in luminosity was measured with a digital luxmeter HER-410 (Steren, Mexico City, Mexico). The colorimetric profile was analyzed using GIMP 2.8.22 free software.

### 2.3. Fabrication and Optimization of Paper-Based Sensors

**Figure 1:**
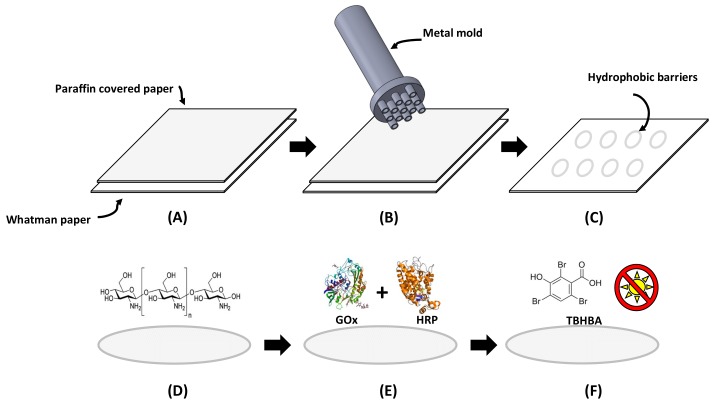
Schematic representation of the paper-based sensor fabrication. From (**A**) to (**C**) the development of the detection zone is shown. (**A**) Paraffin covered paper was attached to Whatman paper, (**B**) a heated metal mold was pressed against papers, and (**C**) defined micro zones with hydrophobic barriers were obtained. Then from (**D**) to (**F**) chemical modification of detection zone is presented: (**D**) chitosan modification, (**E**) the addition of an enzymatic mixture, and (**F**) the addition of TBHBA under dark conditions.

Delimited circular detection zones were created using a stamping process, similar to the methodology for paper devices described by de Tarso-García et al. [28]. A stamp was designed in Solid Works^®^ and manufactured in aluminum to obtain detection zones 3 mm in diameter. The specific dimensions of the metal mold can be consulted in Supplementary File 1. During the fabrication, a piece of Whatman filter paper was dipped into liquid paraffin for 2 s and attached to a piece of non-covered Whatman paper. Then, the aluminum mold was heated at 113 °C and pressed against the waxed paper for 5 s. This resulted in the transfer of paraffin from the waxed paper to the non-covered filter paper and the creation of delimited detection zones with hydrophobic paraffin barriers. The process is schematically represented in Figure 1A–C.

The conditions for the optimal glucose colorimetric assay such as pH and chitosan concentration were evaluated. The detection zones were modified with solutions at 0.25%, 0.5%, 0.75%, 1%, 2%, and 3% (*m*/*v*) of chitosan prepared in acetic acid at 2% (*v*/*v*). Mixtures of GOx (120 U/mL) and HRP (30 U/mL) were prepared in buffer at pH 6.0, 7.0, and 7.4 to investigate different conditions for the enzymatic reaction. The chromogenic agent was prepared with 1.3 mL of PB at pH 7.0, 2.0 mL of TBHBA at 5 mgmL^−1^, and 0.5 mL of 4-AAP at 0.1 M. In order to enhance the sensitivity of the assay, paper-based sensors were prepared by sequentially adding all of the reagents as presented in Figure 1D–F [14]. Firstly, 3 μL of chitosan solution was added to the detection zone and allowed to dry at room temperature. Then 1.0 μL of the enzymatic mixture was spotted on each detection zone and allowed to dry at room temperature. Finally, 2.0 μL of chromophore solution was added on each detection zone under dark conditions. The prepared paper-based sensors were stored at −4 °C in the fridge, avoiding direct exposure to light.

### 2.4. Procedure for Glucose Detection

To verify the ability of the developed paper-based sensor for detection, stock solutions of glucose in PB from 0 to 180 mgdL^−1^ were prepared. This wide range includes the studied glucose concentration in saliva [29]. Given the differences in composition between PB and human saliva, a preliminary test with glucose prepared in artificial saliva was also applied to produce calibration data [30]. In both cases, 2 μL of glucose solution was directly applied to the detection zone prepared as reported in Section 2.3. During detection, the added glucose in the presence of GOx enzyme was catalyzed into gluconic acid and hydrogen peroxide (H_2_O_2_). The released H_2_O_2_ in the presence of HRP acted as an oxidizing agent for 4-APP/TBHBA chromophore, which changed from colorless to a purple-colored product, with an intensity proportional to the glucose content [15]. The intensity in color was recorded with an office scanner at 1200 dpi in order to obtain analytical information through image processing with the Red-Green-Blue (RGB) model for color description [25].

### 2.5. Procedure for Color Detection

The obtained visual results were digitalized with a flatbed office scanner used as a color detector in order to process the information with the RGB model [6,25,31]. The RGB model describes a given color as an additive synthesis of the red (R), green (G), and blue (B) components. Each component is represented on a scale from 0 to 255, when 0 represents no contribution to the color and 255 represents the maximum contribution of the component. Usually in this model, white is represented as the maximum contribution of the components and black is established as the lowest value. Since the enzymatic reaction produces a change in the chromophore from colorless to a purple-colored product, during the calibration the white background of the paper substrate was established as the maximum value for all of the components and the produced color was represented as a reduction of this value. Thus, a lower glucose concentration produced a low intensity color described as high RGB values, and higher glucose concentrations produced high intensity changes represented as low RGB values. In order to achieve an accurate description, the total distribution of the RGB values was considered and the average value was used to create calibration curves forming an inverse relation between mean RGB values and glucose concentration in PBS and artificial saliva.

### 2.6. Selectivity and Stability Test

In addition to glucose detection, the selectivity of the paper-based sensor was evaluated with similar carbohydrates such as sucrose and fructose, as well as NaCl and KCl as interfering species. To investigate the stability of the fabricated devices, the colorimetric response for a glucose sample of 18 mgdL^−1^ was recorded over 10 days.

### 2.7. Validation with Real Samples

A group of three volunteers were recruited to evaluate the performance of the paper-based sensor with real samples. All subjects gave their informed consent for inclusion in the study before the samples were collected. The volunteers reported normal oral hygiene and provided 1 mL of unstimulated saliva, which has been reported as an appropriate specimen for glucose detection [32]. The saliva was collected in Eppendorf tubes and immediately spiked with known concentrations of glucose. Then, 2 μL were directly applied to the detection zone without any pretreatment and recoveries were calculated in order to validate the performance of the paper-based sensor.

### 2.8. Evaluation of Color Perception

The subjectivity of color perception and the impact over accuracy was evaluated with a group of 10 individuals. A semiquantitative screening scale for glucose concentration was provided to the volunteers as well as four different random glucose concentrations (unknown for the participants) in two lighting conditions: 40–360 lux and 560–600 lux. The subject was asked to situate the glucose concentration within a range in the provided scale as well as an estimation of concentration.

## 3. Results and Discussion

### 3.1. Fabrication and Optimization of the Paper-Based Sensor

Paper-based sensors were obtained by a stamping method over a cellulose Whatman substrate. Including the manufacturing services and the raw material, the total cost of the stamp was $60.94 USD. Well-known high-throughput methods for paper-based sensor fabrication can be accessed with a cost from hundreds to thousands of dollars of investment [9], which may be justified for mass production but excessive for prototyping paper devices. In comparison with high-throughput methods, the stamping procedure does not require toxic reagents or complex and expensive machinery. Also, the method provided flexibility of design by CAD programs, which allow a variety of geometries. The fabricated paper-based devices were obtained within 5 s and required a volume as low as 2 μL for the enzymatic assay, even with the achieved resolution at the millimeter scale. Thus, this fabrication method showed advantages such as fast, easy, and environmental friendly fabrication with good resolution at a very low cost.

**Figure 2:**
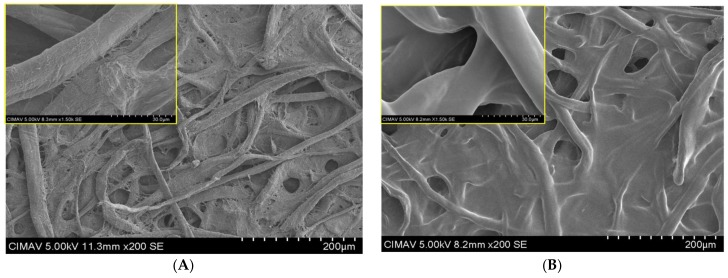
SEM images of the modified paper-based sensor prior to (**A**) and after chitosan modification (**B**) at 2% (*m*/*v*).

Once fabricated, the paper-based sensors were modified with chitosan to obtain a regular spot for detection. The chitosan was applied in concentrations from 0.25 to 3% (*m*/*v*), which created a thin film over the cellulose substrate with a thickness dependent on the concentration. Figure 2 shows the morphology obtained for a paper device prior to and after chitosan modification. Scanning electron microscopy (SEM) revealed the typical porous fibrillar structure of the native cellulose substrate and the presence of a chitosan film covering the cellulose fibers and clogging the porous areas.

**Figure 3:**
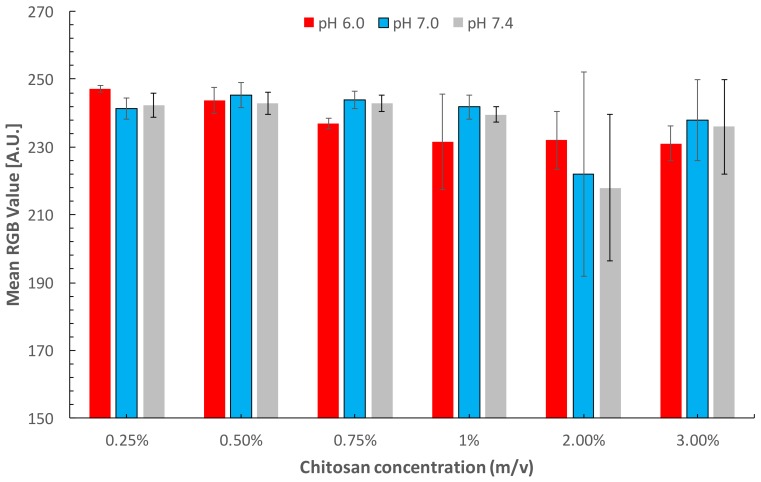
Effect of chitosan concentration and pH over the mean pixel value of a model concentration.

The effect of the chitosan concentration and pH values over the colorimetric response was investigated prior to glucose calibration. The test was conducted with a model concentration of 5.6 mgdL^−1^, which is lower than the current limit of detection (LOD) of commercial glucose colorimetric equipment and is contained within the detection range for glucose concentration in saliva. Figure 3 shows the differences in the average RGB intensity values for the same glucose concentration under different conditions. For concentrations from 0.25 to 75%, the colorimetric outcomes present similar values and low deviation. However, in the detection zones modified with higher chitosan concentration (1–3%), all colorimetric values were enhanced when compared with the low chitosan concentration. The enhanced colorimetric response can be attributed firstly to the delimited detection zones, which increased the reagent concentration in a smaller area as opposite to the dispersion caused by dipping paper without hydrophobic barriers. Moreover, the modification with higher chitosan concentrations contributed to create a biocompatible environment for the enzymatic assay given the high affinity of chitosan for proteins. In general, in spite of showing an enhanced colorimetric outcome, the zones modified with chitosan concentrations higher than 1% showed the highest deviation and poor repeatability in enzymatic assay detection. This was attributed to the hydrophobicity created by the chitosan film in the detection zone. The change in the wettability of cellulose was corroborated by a contact angle test resulting in values of 122.63 ± 5.12 for zones with high chitosan concentrations. The hydrophobic behavior was related to some drawbacks in performance such as irregular reagent distribution, heterogeneous outcome, higher residual standard deviation (R.S.D.), increased analysis time, and high background. Finally, the selected conditions for glucose detection were 0.75% chitosan and pH 6.0 because of the low R.S.D. of 0.29%.

### 3.2. Visual and Quantitative Detection of Glucose in PBS and Artificial Saliva

**Figure 4:**
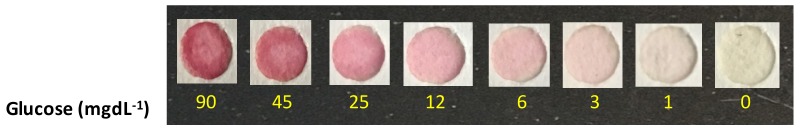
Naked-eye visual scale obtained for glucose detection.

Given the interest to establish a painless method for DM monitoring, sensor devices for the detection of low glucose concentration based on instrumental techniques such as electrochemical detection have been proposed [20,23,33,34,35]. However, only few paper-based sensors for low-cost and reader-free detection have been developed to examine the glucose content in non-invasive body fluids [25,36]. This may be due to the drastically low glucose concentrations found in body fluids compared to blood samples. Unlike the well-recognized physiological range for glucose in blood samples, there is no established range for glucose content in saliva. However, clinical studies using the classic spectroscopic methods have reported concentrations around 1.18 ± 0.675 mgdL^−1^ for healthy individuals and 4.95 ± 2.479 mgdL^−1^ for the diabetic individuals, including values as high as 13.35 mgdL^−1^ ± 6.61 for uncontrolled diabetic patients [37]. Considering these limits, the detection of glucose was carried out by assessing the visual response of the developed paper-based sensor under optimized conditions. Figure 4 shows the visual colorimetric response obtained for the proposed paper-based sensor after increasing the glucose concentration from 0 to 90 mgdL^−1^. The response observed for glucose within the required range for glucose concentration in saliva shows a distinctive difference between values such as 12 mgdL^−1^ (similar to values for uncontrolled diabetic people) and 1 mgdL^−1^ (close to values for healthy individuals). The obtained change in color intensity observed in Figure 4 can be applied as a semiquantitative scale for screening glucose concentration. This approach can be visually examined with the “naked eye”, without the need for external readers or additional requirements, which according to the ASSURED criteria is highly convenient for zones with restricted resources.

**Figure 5:**
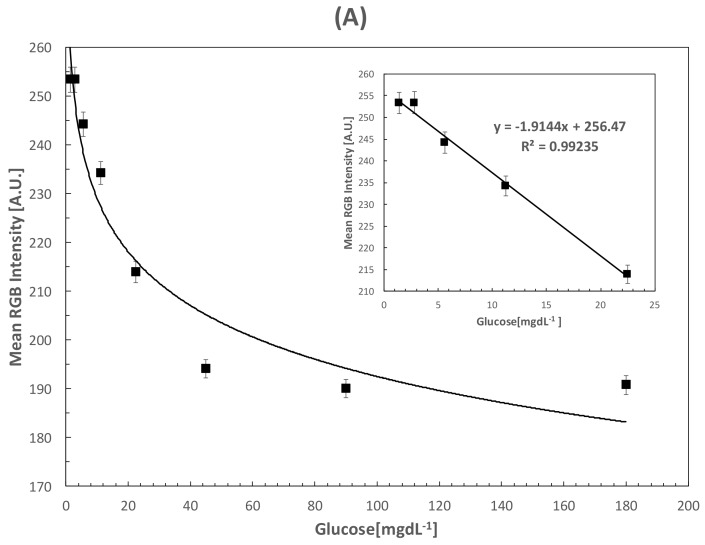

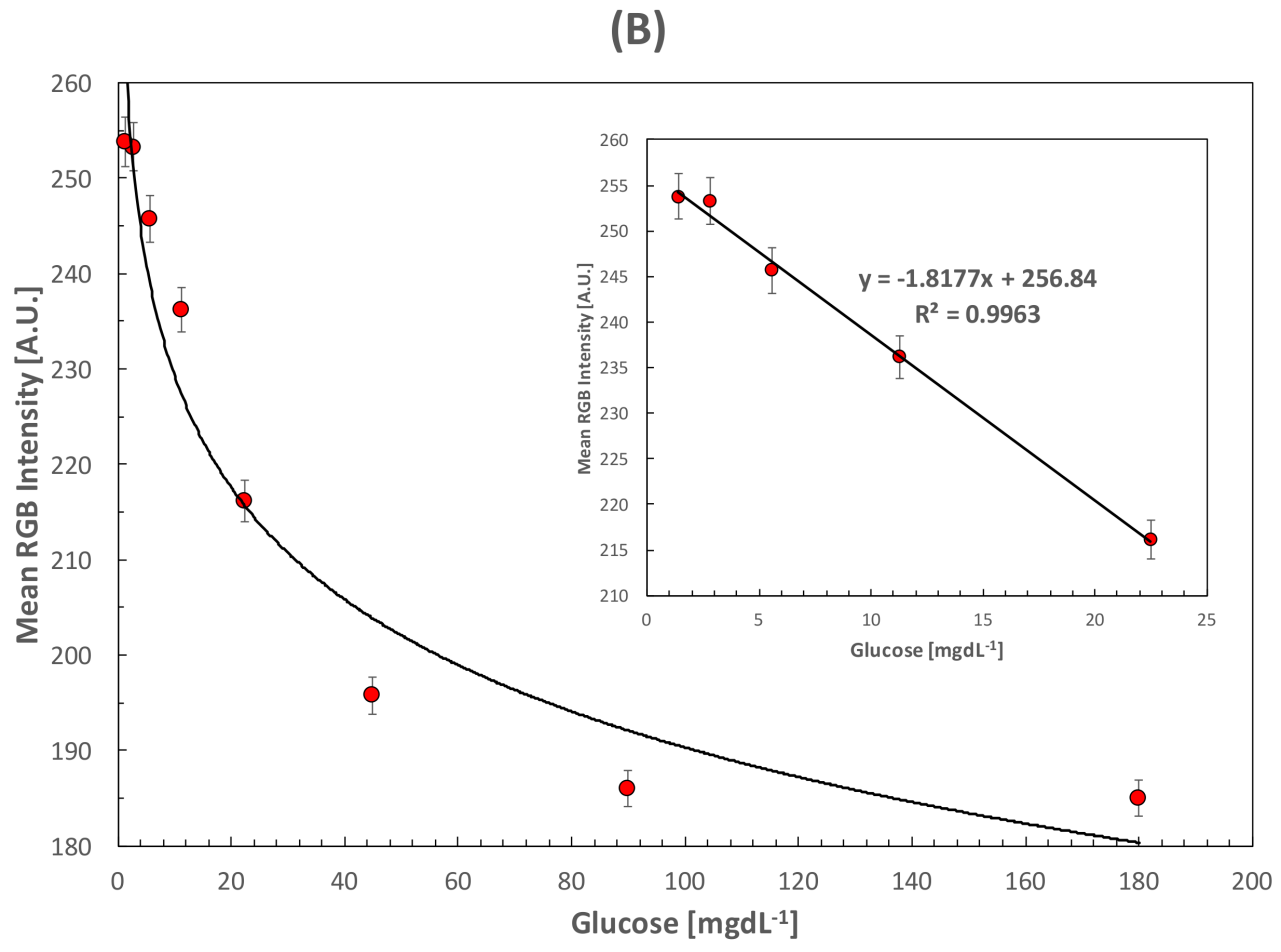
(**A**) Calibration curve obtained for glucose detection in PBS with an inset showing the linear zone; (**B**) Calibration curve obtained for glucose detection in artificial saliva and the augmented linear zone.

The colorimetric results were analyzed with the RGB model in order to obtain the analytical parameters of the paper-based sensor. Increasing glucose concentrations were analyzed in PBS to demonstrate the operational principle of the assay, while artificial saliva was applied to analyze a sample with similar characteristics to human saliva. Figure 5A shows the calibration curve for the colorimetric response of glucose concentrations in PBS within the range from 0 to 180 mgdL^−1^. The curve for glucose quantification showed a linear range from 0 mgdL^−1^ to 22.5 mgdL^−1^ with a calculated LOD of 0.37 mgdL^−1^ (20.86 μM). The inset shows a magnified zone with the linear response of the paper-based sensor, which correlates well with the required glucose concentration in saliva. Figure 5B shows the calibration curve for artificial saliva samples in the range of 0 to 180 mgdL^−1^. Given the similarity in slopes for both curves, the obtained linear range for artificial saliva was similar to that obtained for PBS with a calculated LOD of 0.84 mgdL^−1^ (47 μM). The slight differences can be attributed to fouling effects of artificial saliva components with the developed paper-based sensor; however, the LOD achieved with both assays are among the lowest values for colorimetric paper-based sensors intended for the analysis of glucose in saliva [25]. The reproducibility of the assay measured after 5 days was 3.18%, showing excellent laboratory reproducibility. The analytical figures of merit for both curves, including LOD, limit of quantification (LOQ), sensitivity, goodness of fitting (R^2^) and deviation are presented in Table 1.

### 3.3. Selectivity and Stability Test 

**Figure 6:**
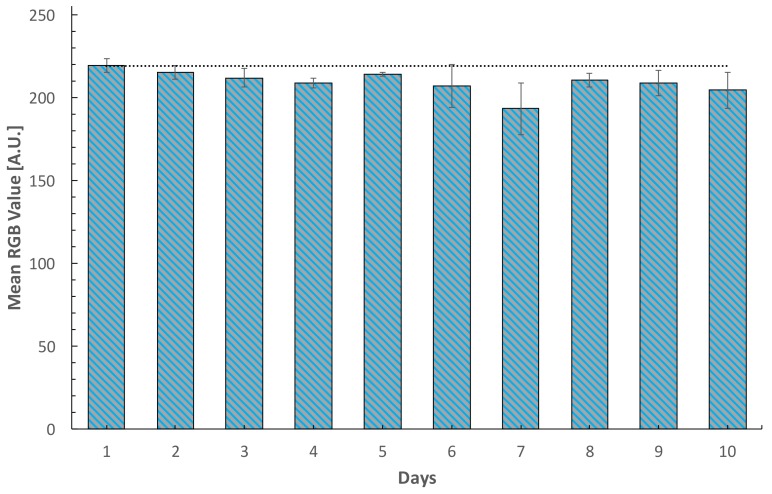
Stability test for fabricated paper-based sensors stored at −4 °C in dark conditions.

The RGB profile of the fabricated devices stored under standard conditions were analyzed for 10 days, as shown in Figure 6. The average deviation registered was 6.32% comparing the RGB profile of the last day with the results of the first day. The stability was achieved with storage conditions of −4 °C in dark conditions, which is easily reachable with a standard fridge. This suggests the potential feasibility of the paper-based sensor in limited resources conditions within an acceptable performance over a period of time. 

**Figure 7:**
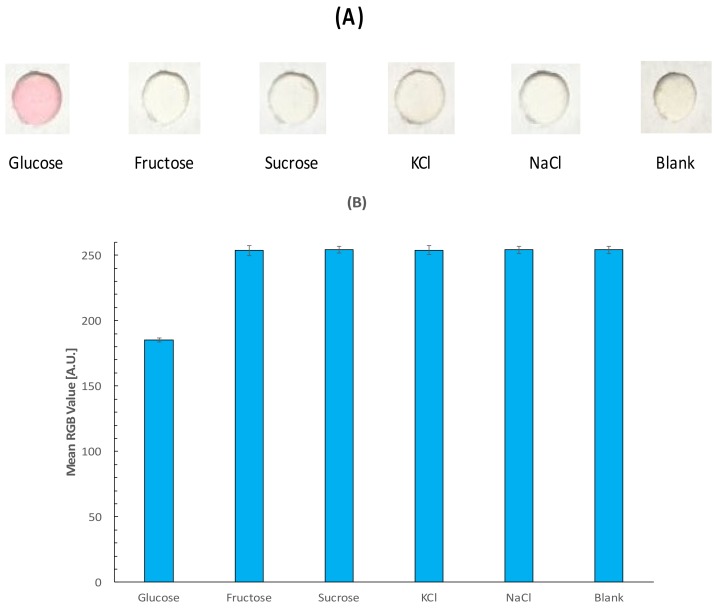
Selectivity test for the fabricated paper-based sensor with colorimetric visual detection (**A**) and RGB value (**B**).

To demonstrate the selectivity of the device, similar carbohydrates such as sucrose and fructose as well as ionic species present in saliva such as Na^+^ and K^+^ were tested in the detection zone. Figure 7 shows the RGB profile of the evaluated samples and a recorded blank of PBS. Considering the inverse relation of the RGB profile and glucose detection, the blank as well as the selected interference species such as fructose, sucrose, KCl and NaCl display a similar behavior close to the maximum value of 255 in the scale, which can be considered as negligible to the detection. 

### 3.4. Detection of Glucose in Human Saliva

To demonstrate the applicability of the paper-based sensor for monitoring real samples, the detection of glucose in human saliva was performed. The developed paper-based sensor was applied to the detection of glucose in saliva samples from three healthy volunteers, who donated 1 mL of unstimulated whole saliva. The accuracy was evaluated through recovery percentages, achieving results from 92 to 114% as shown in Table 2.

Although several approaches to detect glucose have been presented, the screening of non-invasive samples intended for DM monitoring is still in progress. In spite of the low LOD achieved by the nanostructured electrochemical methods, a major drawback presented by these devices is the long pre-processing time before actual measurement is performed. Usually, electrochemical devices suffer from fouling effects over the working electrode and the measurement requires additional preparation steps such as centrifugation of the sample or boiling, which increases the analysis time and demands additional equipment. Since the ASSURED criteria encourages devices for monitoring suitable for regions with limited resources and trained personnel, the paper-based sensor proposed in this work can be applied as an attractive alternative. The recoveries achieved by the developed paper-based sensor are in agreement with those presented by the electrochemical methods with a similar number of samples, suggesting good accuracy compared with these methods. Additionally, they can be operated by untrained personnel since the sample can be easily collected and deposited over the modified detection zone. Also, the stability test suggested that the paper-based devices maintained an appropriate colorimetric performance and only required low-cost infrastructure (standard fridge) for storage.

### 3.5. Evaluation of Color Perception

The influence of lighting conditions over color perception was evaluated using 10 participants. A semiquantitative scale, like that showed in Figure 4, was provided to the participants. The subject was asked to locate a set of four random glucose concentrations within one of the ranges shown in Table 3. Then the subject provided an estimation of the glucose concentration considering the provided scale.

Light conditions of 570–600 can be considered standard for places such as offices, libraries, and classrooms, while lower lux values correspond to bedrooms and hallways. Color perception can be affected by lighting conditions, as revealed by the lower values of recoveries obtained for participants under poor illumination and the most accurate estimation of participants under optimal illumination. This condition should be taking into consideration to obtain accurate estimations. However, a high percentage of participants were able to locate each glucose concentration within its corresponding range. This semiquantitative approach can be designed to establish useful ranges based on meaningful cutoff values such as reported salivary glucose concentrations in healthy and diabetic individuals. Since the presented paper-based device is intended for low-income countries and usually these regions experience limited services, it is feasible to establish a semiquantitative approach for monitoring using the proposed low-cost device.

## 4. Conclusions

We presented the development of a bienzymatic paper-based sensor intended for the detection of glucose in saliva. Delimited detection zones were created by a stamping process and modified with chitosan in order to obtain a uniform spot and a biocompatible environment for the bienzymatic reaction. Naked-eye glucose detection was achieved with TBHBA as a chromophore and the results were corroborated by image processing software. The PBS calibration curve exhibited an LOD of 0.37 mgdL^−1^ with an R.S.D. of 1.69% and an R^2^ of 0.99235, while artificial saliva obtained an LOD of 0.84 mgdL^−1^ with an R.S.D. of 4.33% and an R^2^ of 0.9963. Validation with real samples was performed with saliva, obtaining recoveries from 92 to 114% with a loss of 6.32% after 10 days of storage in standard conditions and a remarkable selectivity among similar compounds. The evaluation of color perception showed a strong influence of lighting conditions, which should be taken into consideration to achieve accurate estimations. However, the device showed good performance as semiquantitative method. The proposed device showed potential as a painless glucose detection method with highly sensitive, facile, low-cost, and device-free operation suitable for highly limited regions.

## Figures and Tables

**Table 1:** Analytical figures of merit for the developed paper-based biosensor.

Matrix	LOD (mgdL^−1^)	LOQ (mgdL^−1^)	Sensitivity (A.U./mg)	R^2^	R.S.D.
Phosphate Buffer	0.37	2.34	1.91	0.99235	1.69
Artificial Saliva	0.84	2.47	1.81	0.9963	4.33

**Table 2:** Results for glucose determination in real samples.

Matrix	Added (mgdL^−1^)	Found (mgdL^−1^)	Recovery (%)	R.S.D. (*n* = 3)
Unstimulated whole saliva	4.0	3.68	92	1.45
	2.8	3.21	114	2.74
	1.8	1.74	96	1.69

**Table 3:** Results of the evaluation of color perception.

Glucose (mg/dL^−1^)	Range (mg/dL^−1^)	570–600 lux			40–360 lux		
		Predicted *n* = 5	Recovery (%)	Selected Range *	Predicted *n* = 5	Recovery (%)	Selected Range *
2.5	1–3	2.7	108	80%	2.91	116.66	80%
4.5	3–6	4.3	95.55	100%	3.58	79.62	100%
9	6–12	8.8	97.77	100%	6.66	74.07	80%
18	12–25	24	133	80%	17.5	92.22	100%

* The percentage of participants that correctly assigned the given concentration to its determined range.

## Supplementary Materials

The following are available online at http://www.mdpi.com/1424-8220/18/4/1071/s1, Supplementary File 1: Mechanical design of metal mold.
