# Supplementary material for: Naked-Eye Detection of Glucose in Saliva with Bienzymatic Paper-Based Sensor
Source: Sensors (Basel). 2018 Apr 3;18(4):1071. doi: 10.3390/s18041071 (PMC5948659; doi:10.3390/s18041071)
Supplement: Supplementary file 1 [file sensors-18-01071-s001.pdf]

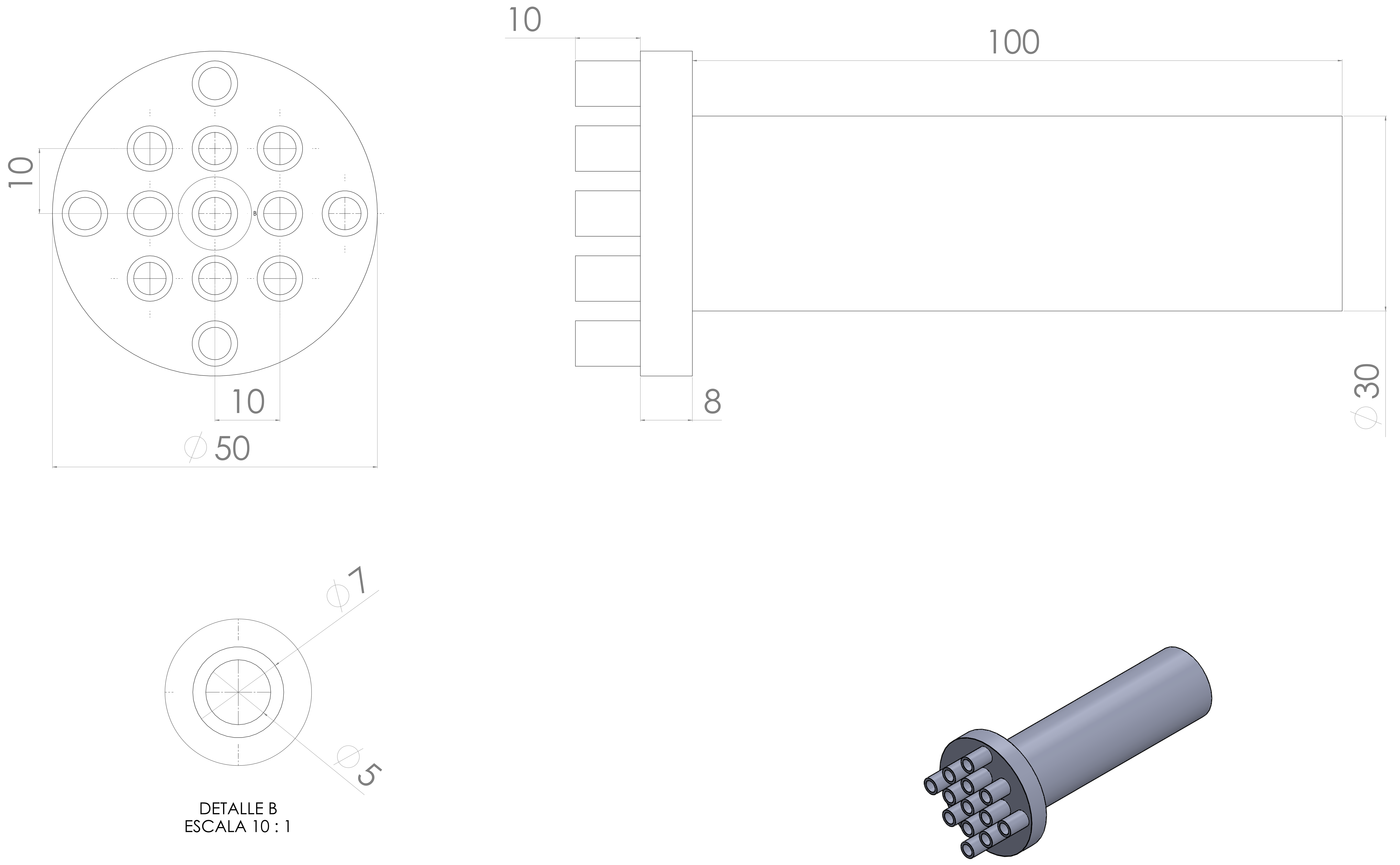

DETALLE B
ESCALA 10 : 1

SI NO SE INDICA LO CONTRARIO: LAS COTAS SE EXPRESAN EN MM ACABADO SUPERFICIAL: TOLERANCIAS: LINEAL: ANGULAR:	ACABADO:	REBARBAR Y ROMPER ARISTAS VIVAS	NO CAMBIE LA ESCALA	REVISIÓN
	NOMBRE	FIRMA	FECHA	TÍTULO:
DIBUJ.				
VERIF.				
APROB.				
FABR.				
CALID.			MATERIAL:	N.º DE DIBUJO nueva pieza
				A0
			PESO:	ESCALA:2:1
				HOJA 1 DE 1
